# Milk Ladder Efficacy and Safety in IgE‐Mediated Cow's Milk Allergy: A Systematic Review and Meta‐Analysis of Controlled Studies

**DOI:** 10.1002/clt2.70122

**Published:** 2025-11-28

**Authors:** Barbara Cuomo, Maria Carmen Verga, Enza D'Auria, Michele Miraglia Del Giudice, Caterina Anania, Fabio Decimo, Giuliana Giannì, Giovanni Cosimo Indirli, Enrica Manca, Filippo Mondì, Valentina Nosratian, Erica Pendezza, Marco Ugo Andrea Sartorio, Mauro Calvani

**Affiliations:** ^1^ Central Operative Unit of Pediatrics and Allergy Center for Children and Adults “Saint Rose” Hospital Viterbo Italy; ^2^ Local Health Unit Salerno Salerno Italy; ^3^ Allergy Unit, Department of Pediatrics Buzzi Children's Hospital Milan Italy; ^4^ Department of Biomedical and Clinical Sciences University of Milan Milan Italy; ^5^ Department of Woman, Child and General and Specialized Surgery University of Campania “Luigi Vanvitelli” Naples Italy; ^6^ Department of Maternal Infantile and Urological Science Sapienza University of Rome Roma Italy; ^7^ Department of Woman, Child and General and Specialized Surgery University of Campania “L. Vanvitelli” Naples Italy; ^8^ Pediatrics Department, Allergology and Immunology Livorno Ospedali Riuniti SIAIP Referent for Emilia‐Romagna Livorno Italy; ^9^ Pediatric Allergology and Immunology Society (SIAIP) Representative for Basilicata Lecce Italy; ^10^ Pediatrics Department Policlinico Riuniti University Hospital of Foggia Foggia Italy; ^11^ IDESP University of Montpellier—INSERM Montpellier France; ^12^ Allergy Center IRCCS Istituto Giannina Gaslini Genoa Italy; ^13^ Pediatric Allergology Unit, Department of Childhood and Developmental Medicine “Fatebenefratelli‐Sacco” Hospital Milan Italy; ^14^ Operative Unit of Pediatrics “S. Camillo‐Forlanini” Hospital Rome Italy

**Keywords:** anaphylaxis, baked milk allergy, desensitization, dietary therapy, epinephrine, milk allergy, milk ladder, oral immunotherapy, tolerance

## Abstract

**Background:**

Milk ladder (ML) is considered a potential therapeutic option for managing cow’s milk allergy (CMA). Although the ML was initially developed to reintroduce cow's milk into the diet for individuals with non‐IgE mediated CMA, it has also recently been used in IgE‐mediated CMA.

**Objective:**

To perform a systematic review and meta‐analysis of the safety and efficacy of ML in children with IgE‐mediated milk allergy.

**Methods:**

We conducted a systematic literature review and meta‐analysis of controlled studies in accordance with PRISMA guidelines. ML safety and ML efficacy compared to a strict avoidance diet and oral immunotherapy in children with IgE mediated CMA was evaluated. Meta‐analysis was performed where ≥ 3 studies reported data. Methodological quality and risk of bias were systematically assessed.

**Results:**

Six controlled studies (two randomized controlled trials and four observational controlled studies) met the inclusion criteria. ML resulted in the development of tolerance in 69% of participants (OR = 4.48; 95% CI = 2.51–8.00), with a significant improvement compared to the elimination diet. The meta‐analysis of four studies showed that ML was 4.5 times more effective than a strict avoidance diet in inducing partial or total tolerance in ITT analysis and 8.4 times in PP analysis. Certainty of evidence was moderate, with low heterogeneity among studies. No significant difference in adverse events, severe systemic allergic reactions, and adrenaline use between the ML and avoidance diet groups were found. Only one study reported comparable efficacy between ML and oral immunotherapy with raw milk (OIT). No difference was found in the total number of mild to moderate adverse reactions between the groups (68.2% and 77.8, respectively; *p* = 0.44). The rates of epinephrine use at home were 14.3% and 11.8%, respectively, without significant difference between ML and OIT groups (*p* = 1).

**Conclusions:**

ML represents a promising therapeutic approach for accelerating tolerance in children with IgE‐mediated CMPA. Further research is needed to clarify milk ladder safety, as well as to identify predictive biomarkers for patient selection.

## 1 Introduction

Food allergy (FA) is a health issue that has been increasing at a global level [1]. Approximately a decade ago, in response to the increasing prevalence and frequent misdiagnosis of food allergies, the National Institute for Health and Clinical Excellence (NICE) was tasked with creating a document to improve the diagnosis and management of these conditions in primary care and community settings [2]. This Guideline particularly emphasized the need to clinically differentiate between non‐IgE mediated and IgE‐mediated expressions of food allergy.

Subsequently, the Milk Allergy in Primary Care Guidelines (MAP) [3], published in 2013, addressed the initial presentation of the clinical expressions of IgE and non‐IgE cow's milk allergy (CMA) in infancy and the on‐going management in primary care of children with confirmed mild‐to‐moderate non‐IgE mediated CMA.

The first “milk ladder” initially appeared as part of MAP, with a 12‐step diet incremental milk dose based on UK diets. [3].

The ML process starts with minimal amounts of less allergic foods and gradually progresses to more allergic options to assess tolerance. The term “ladder” indicates a step‐wise progression from extensively heated to less heated food, with progressively increasing amounts of milk protein as the temperature and timing of heating decreases [4]. The rationale behind the development of this approach is based on the knowledge that cow's milk proteins are modified by heat, though whey‐proteins and casein response to heating differs significantly. Casein (Bos d 8) is a linear epitope and highly heat‐stable (up to 60 min of heating/baking), whereas whey proteins, such as alpha‐lactalbumin (Bos d 4) and beta‐lactoglobulin (Bos d 5), have a conformational structure and heat‐labile, losing allergenicity during extensive heating [4]. Given these differences, the preparation of food products and allergenic changes inherent to the foods in milk ladders can lead to a highly variable delivery of allergenic protein doses [5, 6]. Small amounts of the allergen are initially introduced in a baked form, where cow's milk protein is mixed with wheat flour and extensively heated (e.g., in a biscuit or muffin) for about 20–30 min. Milk protein levels then gradually increase, following a structured approach, which involves progression from extensively heated milk products to those that are less processed, until arriving at unheated milk [3, 7].

The ML is increasingly recognized as a practical and accessible approach. It is a potentially effective home therapy for managing children’s milk allergies and offers a cost‐effective alternative.

Possible benefits of using ladders may include increased food variety or diversity [8], reducing the risks of prolonged or unnecessary milk avoidance. The overall nutrient supply, especially protein and calcium intake, improves with increasing quantity and less processed milk.

A further development of MAP was published in 2017, presenting an internationally applicable diagnostic and therapeutic algorithm (iMAP) [7]. This updated framework categorizes clinical presentations into four categories, delineating criteria for allergen reintroduction tests. It was originally reserved for children with mild to moderate non‐IgE‐mediated allergy, excluding FPIES and EoE. Additionally, the second iteration of the ML underwent simplification and modification, factoring in parameters such as cow’s MP dosage, cooking duration, temperature, matrix effects, and dietary habits across diverse geographical regions. The IMAP comprises a streamlined sequence of six steps.

Since IMAP publication, several countries have adapted the iMAP version and aligned it with their specific nutritional customs, as recently outlined [8]. Although the ML was initially developed to reintroduce cow's milk into the diet for individuals with non‐IgE mediated CMA, it has recently been used in IgE‐mediated CMA [9].

Despite its wide use in clinical practice, there is neither a strictly defined protocol for ML, nor a specified duration for remaining at each step. Given the increased risk associated with IgE‐mediated clinical forms, evaluating the effectiveness and safety of ML compared to other treatments, such as elimination diet or conventional oral immunotherapy (OIT) using raw or baked milk is essential. A systematic review aiming to evaluate the safety and efficacy of dietary advancement therapies (DAT) [10], included milk (ML) and egg (EL) ladders, as well as baked milk (BM‐OIT) and baked egg (BE‐OIT) oral immunotherapy, was recently published. The review included different study designs, including uncontrolled studies. The authors used the GRADE approach to assess the certainty of the evidence, and where possible, they performed meta‐analyses. The results indicated that 58% of patients treated with the ML acquired tolerance, while adverse reactions occurred in 25% of cases, with 2% requiring epinephrine. The authors concluded that there is minimal certainty of evidence supporting DAT safety and they cannot conclude that DAT accelerates tolerance development.

However, the uncontrolled studies included in the systematic review by Anagnostou et al. necessitated using a single‐arm proportional meta‐analysis of the intervention groups and creating control groups supplemented with placebo arms from milk and egg OIT trials. The authors therefore combine data from randomized controlled trials (RCTs), controlled observational studies, and uncontrolled studies with historical controls. The inclusion of uncontrolled studies, at high risk of bias, comparisons between groups that do not belong to the same population, and the pooling of results from studies with different designs introduce methodological flaws.

The Update Cochrane Handbook recommends that non‐randomized studies with very different study design be analyzed separately [11]. This recommendation implies that RCTs and uncontrolled studies should not be combined in a meta‐analysis.

In our review, the selection of studies and the pooling of results are more rigorous, providing a more accurate estimate of the true treatment effect.

Furthermore, new evidence has been published since the meta‐analysis publication.

We conducted a systematic review and meta‐analysis of randomized clinical trials and observational controlled studies to investigate the safety and efficacy of milk ladder.

## 2 Aims

The primary aim of this systematic review (SR) was to evaluate the efficacy of ML in IgE‐mediated milk‐allergic children in favoring the achievement of tolerance. The efficacy is intended as tolerance to fresh raw cow’s milk.

The secondary aim was to evaluate the safety of ML in comparison to other treatments, including the avoidance diet and OIT.

## 3 Materials and Methods

The protocol for this systematic review (SR) has been registered with PROSPERO [12]. Its registration number is CRD42024623796 (accessed on 03.01.2025).

The SR adhered to the Preferred Reporting Items for Systematic Review and Meta‐Analysis Protocols (PRISMA‐P) statement. [13].

### 3.1 Users of the Paper

The present systematic review is intended for hospital and university pediatric allergists, family pediatricians and gastroenterologists.

### 3.2 Setting

The paper mainly addresses Primary Care settings, Hospitals, and University Allergy Centers.

### 3.3 Working Group

The Multidisciplinary Panel (MD Panel and one Registered Dietitian E.P.) worked out the key questions (KQs), discussed evidence, and formulated the conclusions. The MD Panel included specialists in childhood nutrition, family and hospital pediatricians, as well as specialists/experts in allergology, epidemiology, and research methodology.

The group analyzed the results and also contributed to drafting recommendations for the evidence grading using the GRADE method (Grading of Recommendations, Assessment, Development, and Evaluation) [14].

### 3.4 Formulation of Clinical Questions

The clinical questions and related outcomes were identified and developed using the PICO framework, identifying the population, intervention (s), comparison, and outcome (Table 1).

**TABLE 1:** PICO for study inclusion.

Participants (P)	Intervention (I)	Comparison (C)	Outcomes (O)
Inclusion criteria: milkIgE‐mediated allergic children < 18 years	Dietary of increasing the amount of MP in gradually less processed forms	Milk elimination diet or classic oral desensitization treatment	Efficacy of ML on tolerance acquisition, defined as the ability to introduce raw milk into the diet. Safety of ML defined as the anaphylaxis or epinephrine use and other allergic reactions occurred during treatment
Exclusion criteria: Non‐allergic or NON Ig‐mediated allergic children < 18 years	Studies focused on dietary interventions different from ladder scale approach or without at least two steps	No comparison or other kind of control diets	

Abbreviations: ML, milk ladder; MP, mil proteins.

The Panel classified the outcomes as “critical” or “important,” which were considered in the literature review and, subsequently, in the evidence grading.

### 3.5 Literature Search Strategy

A comprehensive search was conducted from January 1, 2000, to December 15, 2024.

We searched the following databases: PubMed, EMBASE, Scopus, CDSR—Cochrane Database of Systematic Reviews, DARE—Database of Abstracts of Review of Effects in Cochrane Reviews, Other Reviews; complementary manual Search was also done on the references of the articles that were retrieved in full. Search terms: cow's milk allergy”[All Fields] OR “food allergy”[All Fields] AND “baked milk”[All Fields] OR “processed milk”[All Fields] OR “cooked milk”[All Fields] OR extensively heated milk”[All Fields] OR “heated milk”[All Fields] AND “ladder”[All Fields] OR “tolerance”[All Fields] OR “desensitization”[All Fields] OR “immunotherapy”[All Fields] OR “safety”[All Fields].

### 3.6 Inclusion Criteria

– Publication date: for SRs: last 10 years; for studies: from the inception to December 15, 2024;
– Language of publication: English;
– Type of studies: Randomized Controlled Trials (RCTs) and controlled observational Studies (retrospective case‐control studies, prospective cohort studies)
– Population: children under 18 years of age, children were considered eligible if diagnosed with cow's milk allergy
– Intervention: We considered milk Ladder protocols which include at least two steps, with increasing amount of milk proteins and different milk products
– Outcomes:

- Achievement of partial or complete tolerance to MPs; partial tolerance refers to the ability to consume limited amounts of milk without reactions, while complete tolerance indicates unrestricted intake of milk and dairy products;

- Adverse reactions, such as anaphylaxis or epinephrine use and other allergic reactions (e.g., skin, laryngeal, respiratory, gastrointestinal, and cardiovascular symptoms)

– Relevance to the research question
– Methodological validity: assessed according to the minimum criteria described in the “Analysis of Scientific Evidence” section.

### 3.7 Exclusion Criteria

– Abstracts, case report, case‐series, narrative review
– Studies on dietary therapy that differed from ML based on at least two steps
– Observational uncontrolled studies, e.g., studies that did not compare the treatment under evaluation (ladder) with the outcomes of control groups
– Controlled trials that did not compare the treatment under evaluation with the outcomes of control groups treated with OIT or avoidance diet

### 3.8 Study Selection

A preliminary selection was conducted based on titles and abstracts, excluding studies available only as abstracts. The full texts of studies considered potentially relevant or requiring further information to determine relevance were obtained.

Two independent researchers (M.C. and F.M.) screened the databases. The references were imported into citation management software (EndNote 20.2.1) for initial duplicate removal. They independently screened the search string, reviewed all abstracts, and agreed on which full‐text articles to retrieve for assessing potentially eligible studies. Disagreements were resolved through discussion, and, if required, in cases of incongruence, a third reviewer (B.C.) was responsible for mediating a discussion and consequential decisions.

We contacted the study authors for data clarification and additional information if necessary.

The eligible SRs and studies were further assessed for their overall methodological quality, including how missing data were handled.

### 3.9 Analysis of Scientific Evidence

Two authors (B.C. and M.C.V.) independently assessed the included studies for the risk of bias. The reviewers were blinded to each other’s assessments. They resolved any disagreement through discussion and, if necessary, involving a third author (M.C.).

The SRs were analyzed using the validated AMSTAR 2 (Assessment of Multiple Systematic Reviews) tool [15]. The minimum validity criterion was an overall assessment of low methodological quality.

The quality of randomized studies was assessed using the Cochrane tool for assessing risk of bias [16].

For observational studies, the Newcastle‐Ottawa Scale (NOS) [17] was used for cohort, case‐control, and cross‐sectional studies. Studies were classified as low quality (0–4 points), medium quality (5–8 points), and high quality (9–12 points), ensuring a transparent and fair judgment process.

Bias and confounding factors were considered when assessing study quality.

### 3.10 Data Extraction and Management

At least two authors independently extracted data using a predefined scheme based on the PRISMA [13] checklist criteria and verified its accuracy.

All data were entered into Review Manager 5 for RCTs and meta‐analyses assessment (Review Manager, 2014). [15].

### 3.11 Effect Size

Dichotomous variables—Outcomes were calculated as Relative Risk (RR) for prospective studies or Odds Ratio (OR) for retrospective studies, with 95% confidence intervals (CI).

Continuous variables—Mean differences (MD) with 95% CI were calculated for continuous variables.

### 3.12 Missing Data

Loss to follow‐up and compliance with any interventions were assessed in all the studies included.

The results obtained were analyzed, as far as possible, by intention‐to‐treat analysis and per protocol. Only available data were used; no other data were entered when data were missing.

### 3.13 Evaluation of Heterogeneity

The heterogeneity across the studies was also assessed.

Methodological heterogeneity was assessed by examining the risk of bias. In contrast, clinical heterogeneity was evaluated by exploring similarities and differences across the studies regarding types of participants, interventions, and outcomes. Effect size and direction were calculated, and tau^2^, *I* ^2^, and Chi^2^ statistical methods were used to quantify the level of statistical heterogeneity across the studies in each analysis.

Heterogeneity was assessed via visual inspection of the forest plot, using the Chi^2^‐test (significant if *p* < 0.10) and *I* ^2^ statistic (heterogeneity considered significant if *I* ^2^ > 60%). Thresholds for the interpretation of the *I* ^2^ statistic can be misleading, since the importance of inconsistency depends on several factors. A rough guide to interpretation in the context of meta‐analyses of randomized trials is as follows:0%–40%: might not be important; 30%–60%: may represent moderate heterogeneity*; 50%–90%: may represent substantial heterogeneity*; 75%–100%: considerable heterogeneity(the categories overlap, as interpretation depends on both contextual factors and clinical coherence).

Special caution was taken when interpreting results with high heterogeneity levels.

### 3.14 Data Synthesis

Meta‐analyses were conducted when data could be aggregated. Separate analyses were carried out for observational studies and RCTs.

In case of significant statistical heterogeneity, data were combined using the random‐effects model. The Mantel‐Haenszel method was applied for dichotomous results, and inverse variance was used for continuous results.

### 3.15 Software

RevMan 5.4.115 was used to evaluate the methodological quality of RCTs, meta‐analyses, and related figures [15].

The GRADE pro GDT software, developed by the GRADE Working Group, assessed the overall quality of the collected evidence and relevant tables [18].

## 4 Results

**FIGURE 1:**
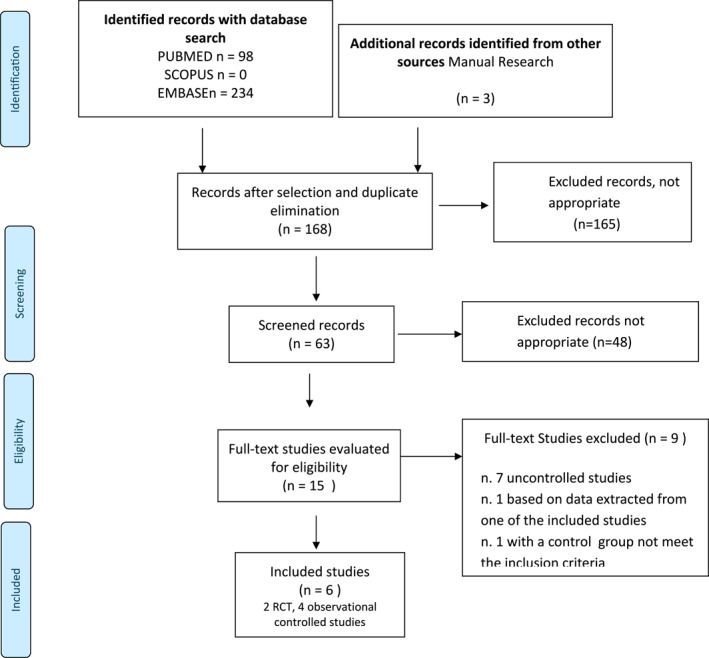
Studies search flow diagram.

The selection and inclusion process of the studies is described in the PRISMA Statement Flowchart (Figure 1).

In total, 332 articles were retrieved. After screening, one SR and 15 studies were identified as relevant. Evaluating the references of the most pertinent studies did not lead to the identification of additional articles. The full texts were assessed for eligibility. The identified SR was excluded due to critically low methodological quality. Nine out of 15 were non‐controlled studies or included control groups that differed from those specified in the inclusion criteria of this review (Figure 1).

In total, we included six studies in this systematic review [19, 20, 21, 22, 23, 24]:two RCTs, by Amat et al. [19] and Esmaeilzadeh et al. [20], were of high [19] and moderate [20] quality, respectively; three cohort studies, Nowak‐Węgrzyn et al. [21], Kim et al. [22], and Trujillo et al. [24], were of high [21], and moderate [22, 24] quality, respectively, while the case‐control study, by Efron et al. [23], of moderate quality.

Tables 2 and 3 show the basic characteristics of included and excluded studies, respectively.

**TABLE 2:** Milk ladder controlled studies in IGE‐mediated allergy.

Author/year Country	Type of study	Sample size	Medium age	Follow‐up	Clinical aspects of the allergy	SPT/IgEs positivity	Diagnosis of food allergy	Ladder protocol	Control group	Outcome	Results	Safety	Funding
Trujillo [24] 2024 Ireland‐Spain	Historical cohort	371 AG: 171 CG: 200 Drop out: 29 AG 0 CG	AG: 12 months CG: 5 months	Not reported	Different severity of immediate allergic reactions. Severe allergic reactions were included too	100%	Not reported	Twelve steps according to MAP milk ladder. Applied to Irish children	Spanish children in strict elimination diet for milk and dairy products	Successful reintroduction of cow's MP	Tolerant to raw milk: AG: 86.6% CG: 61% *p* = 0.001 Time of tolerance: AG: 12.5 months CG: 21 months *p* < 0.001	AEs AG: 32 (18.7%; 95% CI: 80.6–90.9) CG: 106 (53%; 95% CI: 54.1–67.5) *p* < 0.001 Anaphylaxis: AG: 3 (1.8%; 95% CI: 0.6–5.0) CG: 34 (17%; 95% CI: 12.4–22.8) *p* < 0.001	From the National Dairy Council and Dairy Research, Ireland.
Nowak‐Węgrzyn [21] 2018 USA	Prospective	170 AG: 136 CG: 34 Drop out: 32 AG 12 CG	6.6 years	36 months	Not reported	100%	Positive SPT or IgEs with a convincing clinical history or elevated IgEs value over positive predictive values or DBPCMC in the previous 2 years	Four ladder steps under medically controlled OFC increases. Steps: Muffin, pizza, rice pudding, raw milk. They randomized reactive children to different degrees of milk 1:1 to increases every 12 months (AG12: 44 subjects) or 6 months (AG6: 41 subjects).	Children in strict elimination diet for milk and dairy products whose parents chose not to participate in the active intervention arm.	Defined odds ratio for progression to tolerance in 6 versus 12 months scale group and degree of developed tolerance over 3 years	No significant difference observed between the 6 and 12‐month escalations. Tolerant to raw milk: AG: 41 (48.2%; AG6: 61% and AG12: 73%) CG: None	AEs: 35% of AG occurred during the first baseline‐OFC or escalation‐OFCs epinephrine used: 22 (17.5%) of AG. No reactions at home required epinephrine; none developed EoE	AI 44236 from the NIAID and in part by UL1 TR‐000067 from the National Center for Advancing Translational Sciences (NCATS), a component of the National Institutes of Health (NIH).
Efron [23] 2018 Israel	Retrospective	110 AG:43 CG: 67 Drop out: Five from AG (11.6%)	21 months	71 months	Gastrointestinal, cutaneous, and respiratory symptoms. Severe allergic reactions were not included	100%	Positive OFC or positive SPT or IgEs with a value above 95% CI for age with a convincing clinical history	Five ladder steps under medically controlled OFC every 3 months. Steps: frying or baking, pancake, toast or pizza, yoghurt, raw milk.	Strict elimination diet for milk and dairy products	Efficacy and safety of structured gradual exposure protocol with heated and baked milk on tolerance acquisition	Tolerant to raw milk: AG: 86% CG: 52% *p* = 0.003 Time of tolerance: AG: 36 months (95% CI, 34.5–49.7) CG: 98 months (95% CI, 82.4–114.1) *p* < 0.001	AEs AG: 16 (37%) of had adverse reactions, three during the initial OFC, mostly mild or moderate. CG: 0 epinephrine used: AG: 2 of at home. CG 0	N.A.
Esmaeilzadeh [20] 2018 Iran	Prospective RCT	84 AG: 42 CG: 42 Drop out: 0	1.7 years (6 months‐3 years)	12 months	Gastrointestinal, cutaneous, respiratory or systemic allergic reactions were included	100%	Positive history of IgE‐mediated milk allergy plus positive SPT (more than > 8 mm) or positive IgEs (> 5 kUA/L in children < 2 years or > 15 kUA/L for those > 2 years)	Two steps at home every 6 months. Steps: Muffin with 1.3 g MP (0.65 g for children < 1 year), baked cheese in pizza (4.6 g MP), raw milk OFC with 240 mL (or other products with 8–10 g skim milk like yoghurt)	Strict elimination diet for milk and dairy products	Efficacy of adding baked milk to diet on accelerating tolerance acquisition	Tolerance at 12 months: AG: 88.1% (37/42) CG: 66.7% (28/42) *p* = 0.018	Not reported	N.A.
Amat [19] 2017 France	Prospective RCT	41: AG: 23 CG: 18 Drop out: 4	6 years old	18 months	Different severity of immediate allergic reactions	100%	Positive DBPCMC and IgEs ≥ 0.35 kU/L	Three steps at home every 15 days. Steps: Extensively heated baked milk (Véritable Petit Beurre then plus Prince Tout Choco until 210 mg of MP), half‐heated baked milk (Kinder Barrè then Kinder Surprise until 1970 mg of MP), raw milk (until 2720 mg of MP)	Oral desensitization with raw milk. Schedule steps under medically controlled OFC every 5 weeks for 5 months	Efficacy (defined as tolerated 2720 mg of MP) and safety of two OIT protocols	There was no difference between arms. Tolerant to raw milk: 15 (36.6%) partially tolerant: 11 (26.8%), non‐tolerant: 15 (36.6%). *p* = 0.18 There was no difference in the gain of tolerance threshold *p* = 0.24	AEs AG: 24 CG: 16 *p*: 0 *p*.44 Epinephrine used: 5 (13.5%) at home Among which AG: 3 CG: 2 *p* = 1	N.A.
Kim [22] 2011 USA	Prospective case‐control	148 AG: 88 (65 treated with ladder + 23 reacted to baked) CG: 60 Drop out: 11	6.6 years	37 months	Different severity of immediate allergic reactions	100%	Positive SPT or sIgEs and history of an allergic reaction to milk within 6 months; or sIgE levels or SPT > 95% predictive for clinical reactivity (> 5 kUA/L in children < 2 years or > 15 kUA/L for those > 2 years, SPT ≥ 8 mm)	Two ladder steps under medical‐controlled OFC every 6 months Muffin with 1.3 g MP, baked cheese in pizza (4.6 g MP), raw milk with 240 mL (or other products with 8–10 g skim milk like yoghurt)	Strict elimination diet for milk and dairy products	Baked‐milk–tolerant children would benefit from improving nutrition, dietary variety, and tolerance development	Tolerant to raw milk AG: 41/88 (47%) CG: 13/60 (22%) *p* < 0.04 Partially tolerant AG: 11/88 (26.8%) CG: 13/60 (22%), non‐ tolerant: AG: 26 (30%). CG: 34 (56%).	Five mild‐moderate anaphylaxis during challenges. Compared data for anaphylaxis were not reported. EoE: 2 (3.1%) in the AG, 5 (8.3%) in CG	AI 44236 from the NIAID and in part by Grant Number CTSA ULI RR 029887 from the National Center for Research Resources (NCRR), a component of the National Institutes of Health (NIH).

Abbreviations: AEs, adverse reactions; AG, active group; AG6 and AG12, active group with an increase every 6 months or 12 months; CG, control group; CI, confidence interval; DBPCMC, double‐blind placebo‐controlled milk challenge; EoE, eosinophilicesophagitis; IgEs, specific IgE; ITT, intention to treat group; MP, milk proteins; OFC, oral food challenge; OITs, oral immunotherapy treatments; RCT, randomized controlled trial; SPT, skin prick test; vs, versus.

**TABLE 3:** Milk ladder uncontrolled studies in IgE mediated allergy.

Author/year Country	Type of study	Sample size	Medium age	Follow‐up	Clinical aspects of the allergy	SPT/IgEs positivity	Diagnosis of food allergy	Ladder protocol	Control group	Outcome	Results	Safety	Reason for exclusion
Cerecedo [25] 2024 Spain	Not clearly reported	AG: 26 drop out: 2 Withdrawal: 1 (11.5%)	Supplementary material not yet published	Efficacy data not yet published	Supplementary material not yet published	100%	DBPCFC	Four ladder steps every 3 months. Steps: CM biscuits/muffin, panecakes/croquettes, heated cheese, fermented milk, fresh milk.	—	Evaluate the feasibility of the milk ladder in IgE‐mediated CM allergy	Efficacy data not yet published (extracted 84.6% completed the ladder therapy)	AEs: 13.6% of total OFC, 36.4% of patients (8/22). Epinephrine use: 5 in 4 patients	Not controlled study
Gallagher [26] 2024 Ireland	Retrospective	AG: 36 affected by anaphylaxis	12.22 months	12.5 months	IgE‐mediated with symptoms of anaphylaxis at diagnosis	100%	Clinical history Positive SPT or sIgE	Twelve steps according to MAP milk ladder	—	Focus on the use of food ladders in children with anaphylaxis to egg or milk	28 (77.8%) completed the ladder therapy	AEs: 50% Anaphylaxis: 1 (2.7%)	Data extracted from Heng's study 2023 [ ]
Chomyn [27] 2024 Canada	Not clearly reported	109 30 milk ladder 63 egg ladder 16 both ladders AG: 53 Drop out: 10 (18.9%)	38 months	3–6–12 months	IgE‐mediated, independent from the severity of the reaction	Not reported	Allergist‐diagnosed allergy	Four Canadian's ladder steps. Steps: Muffin, pancake, pizza, boiled milk, cheese, yogurt, fresh milk.	—	Safety and effectiveness data for Canadian adaptation of the milk and egg ladders	Not clearly reported for each steps	AEs: Not reported. Anaphylaxis: Not for ML (3.2% for egg ladder)	Not controlled study
Cronin [28] 2023 Ireland	Retrospective	82 (13 in PC, 69 in TC)	8.5 months In PC 11.14 months in TC	36 months	Mainly either cutaneous symptoms or gastrointestinal symptoms	100%	Not reported	Twelve steps according to MAP milk ladder	—	Safety and effectiveness of the use of the milk ladder for children with IgE‐mediated CM allergy	Tolerance at step 12 PC: 85% meantime 12.7 months TC: 83% in the meantime, 15.5 months	AEs PC: 46.2% TC: 46.4% Anaphylaxis: 2, one in each cohort.	Not controlled study
Ah Heng [29] 2023 Ireland	Retrospective	171	12 months	12.5 months	IgE‐mediated, independent from the severity of the reaction	100%	Clinical history Positive SPT or sIgE	Twelve steps according to MAP milk ladder	—	Introduction of more than 150 mL of milk (4.5 g of proteins) without symptoms	148 (86.6%) completed the ladder therapy	AEs: 42.7% Anaphylaxis: 3 (1.8%)	Not controlled study
D'Art [30] 2022 Ireland	RCT prospective	60 AG: 40 CG: 20 Drop out: 3	7.5 months	6 and 12 months	IgE‐mediated, independent from the severity of the reaction	100%	Clinical history Positive SPT	Twelve steps according to MAP milk ladder. From an initial dose of 0.1 mL of baked milk at the hospital, then at home	Children start with a ladder scale at home (without an initial dose under medical supervision)	Evaluate the impact on ladder progression after a supervised, single low‐dose exposure	At 6 months Step 6 AG: 73% CG: 50% *p* = 0.48 Step 12 AG: 30% CG: 10% *p* = 0.49 At 12 months: Step 6 AG: 86% CG: 79% *p* < 0.05 Step 12 AG: 65% CG: 35% *p* = 0.03	Three severe adverse events occurred; more frequently, there were mild reactions. None required epinephrine.	Control group not meet the inclusion criteria
Ball [31] 2019 UK	Retrospective	86 Dropouts = 7 (33%)	13 months	12–16 months	Cutaneous and/or gastrointestinal symptoms and SPT < 8 mm	100%	Clinical history and positive SPT	Four ladder steps at home every 4–6 months. Steps: CM biscuits, baked foods, fermented or heated cheese or heated milk, uncooked cheese or uncooked non‐fermented dessert or fresh milk.	—	Safety, efficacy and compliance of the ladder program	Step 4: 90.7%	AEs: 80% Anaphylaxis 0	Not controlled study
Dunlop [32] 2018 USA	Retrospective	187	6.8 years	49 months	Not reported	100%	Not reported	Five steps every 2–3 months at the hospital or home. Steps: Baked milk (cake or muffin), pancakes and waffles, baked cheese, uncooked dairy products, fresh milk.	—	Give information on long‐term follow‐up from the introduction of cooked milk	Tolerant to raw milk 43% Partially tolerant 30% Non‐tolerant: 28%.	AEs: 92%, EoE: 6 Anaphylaxis: 8.	Not controlled study
Weinbrand‐Goichberg [33] 2017 Israel	Prospective	85 Drop out 3	5.2 years	29 months	Not reported	100%	allergic reaction within 6 months before or sIgE levels or SPT greater than 95% of the predicted value	Two steps every 6–9 months at hospital. Steps: Baked milk (muffin, cake), backed cheese (pastry or pizza), uncooked milk (yoghurt or skim milk)	—	Prospectively evaluate the long‐term safety and natural course of introducing processed milk in allergic children's diet	Tolerant to raw milk 31% Partially tolerant 19% Non‐tolerant: 29%.	Anaphylaxis: 3	Not controlled study

Abbreviations: AEs, adverse reactions; AG, active group; CG, control group; CM, cow's milk; DI, initial dose; EoE, eosinophilicesophagitis; FAQL, food allergy quality of life; OFC, oral food challenge; PC, primary care cohort; RCT, randomized controlled trial; sIgE, specific IgE; SGEP, structured gradual exposure protocol; SPT, skin prick test; TC, tertiary care cohort.

### 4.1 Milk Ladder Effectiveness

Six studies [19, 20, 21, 22, 23, 24] focused on the efficacy of ML in IgE‐mediated allergy (Results in Table 2). Amat et al. [19] and Esmaeilzadeh et al. [20] enrolled allergic children between 7.5 months and 7 years old who received cow's MP with an ML scale. Data were compared to those from children who had an elimination diet [20] or underwent classic oral desensitization [19].

Amat et al. [19] enrolled children between 3 and 10 years old with a cow’s milk allergy, confirmed by a positive DBPCFC. At the enrollment, the children were randomly assigned to two groups using a random number generator.

Children (*n* = 18) included in the first group, called “high risk” (raw milk), underwent classic oral desensitization in a build‐up regimen with fresh cow's milk. Tolerated doses were administered daily at home while up‐dosing was performed under medical control. The final goal was a tolerance of 2720 mg (around 200 mL) of cow's milk proteins in 5 months.

Children (*n* = 23) of the second group, called “low risk” (processed milk), were invited to assume daily products available in the commerce, like biscuits that contain baked cow's milk, then ones with less cooked milk such as cow's milk bars, and finally fresh milk. The amounts of cow's MP was gradually increased every 2 weeks at home. The achieved dose of 210 mg of baked milk proteins permits the switch to half‐heated milk products, and the reached dose of 1970 mg of less cooked MP permits the passage to fresh milk. The goal was to achieve 2720 mg of fresh cow's MP tolerance within 9 months.

At the end of the study, 15 patients were tolerant (36.6%), 11 were partially tolerant (26.8%) with a median threshold of tolerance of 697 mg (27.2–2550 mg), and 15 children (36.6%) were non‐responders. The study did not report differences in the cow's MP quantity reached between the two groups.

Esmaeilzadeh et al. [20] aimed to evaluate if the introduction of baked milk in the diet of children with IgE CMA accelerates tolerance achievement. Eighty‐four children tolerant to baked milk contained in a muffin were asked to continue to assume this dose (1.3 g of baked milk's proteins) for 6 months and then to consume baked cheese in the form of pizza for another 6 months. The control group was strictly instructed to avoid milk products for 1 year. At the end of the study period, 88.1% of the treated patients and 66.7% of those in the control group had tolerance to unheated milk (*p* value = 0.018). Among children with a history of anaphylaxis, 41.1% of treated cases achieved tolerance, whereas none in the control group did.

In other prospective studies, Nowak‐Węgrzyn et al. [21]and Kim et al. [22] compared data from the ML group with patients on a milk‐elimination diet. The prospective study by Nowak‐Węgrzynet al. [21] enrolled children with a history of adverse reactions to cow's milk or a positive DBPCFC performed in the previous 2 years, associated with positive allergic tests. Some children had positive allergic tests performed earlier but remained positive within the last 6 months before enrollment or had specific IgEs or skin prick tests predictive of an adverse reaction (> 14 kU/L *e* > 10 mm, respectively). The patients underwent an OFC every 2 weeks with foods containing an increased dose of cow's milk proteins, firstly baked and then less processed. When a reaction appeared, tests were interrupted, and patients were sent home with directions on how to consume the last tolerated food regularly at home. Children who tolerated heated milk were divided into two groups. Children from the first one were called every 6 months to up‐build doses, and the second one was called every 12 months. They proceeded towards the OFC with foods containing less cooked milk up to fresh milk. The principal goal was to evaluate the degree of developed tolerance between the two groups during the 36 months of the study. The results of both groups were compared to a control group of tolerant children who were given heated milk and had the same initial characteristics but refused to proceed with the ML scale and continued with the elimination diet. Forty‐eight percent (41 patients) of both active groups of 136 enrolled patients reached the final goal and developed tolerance independently of the ML scale progression speed (6 vs. 12 months). In contrast, no control group subject resulted in tolerance at the final OFC.

Kim et al. [22] followed a two‐step ML: it started with baked milk in muffins and, after six or more months, introduced a challenge with baked cheese (pizza) products. Finally, after 6 or more months, children were tested with fresh milk. The controls were 60 children retrospectively gathered and matched for age, sex, and specific IgE value who fulfilled the inclusion criteria. Tolerance was achieved in 59% of the active group and 22% of the control group.

Finally, we have included two controlled retrospective studies, Efron et al. [23] and Trujillo et al. [24], which aimed to evaluate ML efficacy. The former [23] used a ML scale of 5 steps from baked to fresh milk. Data from the active group (43 children) were compared to children treated with a strict avoidance diet until 4 years of age (*N*: 67). Results showed a faster acquisition of tolerance in the ML‐treated group, 36 months, compared to 98 months in the control group. The latter [24]employed a ML scale of 12 steps according to the MAP Milk Ladder. The active group (32 children) showed a higher number of subjects who developed tolerance to raw milk compared to the control group (respectively 86.6% and 61%; *p* = 0.001), and in a shorter time (respectively 12.5 months vs. 21; *p* = 0.001). Of note, a lower value of whole milk‐specific IgE was associated with successful treatments (15.84 kIU/mL, 95% CI: 10.95–20.72 kIU/mL vs. 37.22 kIU/mL, 95% CI: 18.04–56.4 kIU/mL, *p* < 0.01) in the ladder group.

### 4.2 Safety

Four included studies [19, 22, 23, 24]evaluated ML safety outcomes, whereas two studies [20, 21]reported safety without this specific endpoint (Table 2). It is worth of note that 4 out of 6 studies the first introduction dose as an oral food challenge in the Hospital [20, 21, 22, 23], meanwhile, in the remaining two studies, the first dose was administered and then implemented at home with different schemes.

In the study by Amat [19] three steps at home every 15 days were employed, while in the study by Esmadeilzah et al. [20] patients in the case group were asked to consume baked milk in the form of a muffin for 6 months and then to consume baked cheese in the form of pizza for another 6 months.

Overall, the studies show that most cases tolerated the gradual home reintroduction of cooked milk (ML scale). Indeed, patients reported mild to moderate allergic symptoms, which generally did not lead to the discontinuation of the reintroduction process. Adrenaline was used both at the emergency department and at home. Generalized symptoms were not always attributed to the reintroduction process, but also to accidental food exposure. Prospective studies reported epinephrine use in up to 17.5% of hospital challenges [21] and 3/23 (14.3%) children at home [19]. Kim et al. [22] reported that 6/89 (6.7%) had mild‐to‐moderate anaphylaxis during 8/172 (4.6%) challenges. Other adverse reactions, apart from anaphylaxis, were primarily reported in the retrospective study by Efron et al. [23] 16 children (37%) in the intervention group had an allergic reaction, mostly mild or moderate, three of these occurred during the initial observed OFC with extensively heated baked milk. However, two adverse reactions were treated with epinephrine. Results from Trujillo et al. [24], the most recent retrospective study, showed that among the milk avoidance group accidental exposure to milk unexpectedly occurred in 34/106 (32%) cases, resulting in an anaphylactic reaction. Meanwhile, in the active group (32 patients), 3/32 (9.3%) had accidentally consumed a less extensively heated milk product and experienced anaphylaxis during the treatment ML period. Most interesting is the comparison with the results from the study by Amat et al. [19], which included one group treated with ML and another with classic OIT. There were no differences in the number or severity of adverse reactions (RR = 0.85; 95% CI = 0.50–1.46). One subject per group abandoned the study due to mild to moderate adverse reactions; adrenaline was used in five cases—2/18 (11.8%) in the “high risk” group (OIT) and 3/23 (14.3%) in “low risk” group (ML) (RR = 1.17; 95% CI = 0.22–6.30), respectively. Two of these patients stopped the treatment.

### 4.3 Meta Analysis‐Results

The meta‐analysis assessing the efficacy of ML in achieving partial and total tolerance included four observational studies [19, 20, 21, 22], analyzing 438 patients. Results showed a significant overall effect, Odds Ratio = 4.48 (95% CI = 2.51, 8.00; *p* < 0.0001) in the Intention to treat analysis, favoring the ML over the elimination diet. Heterogeneity was not significant (*I* ^2^ = 43%; *p* = 0.15; Figure 2.1). Tolerance occurred in 69% of patients undergoing ML treatment. Results improved in the per‐protocol analysis (OR = 8.41–95% CI = 2.74, 25.76; *p* = 0.0002). Heterogeneity was significant (*I* ^2^ = 78%; *p* = 0.004; Figure 2.2). Regarding safety, the pooled Odds Ratios indicated no significant difference in adverse events, severe systemic allergic reactions, and adrenaline use between the ML and elimination diet groups (Figure 2.3).

**FIGURE 2:**
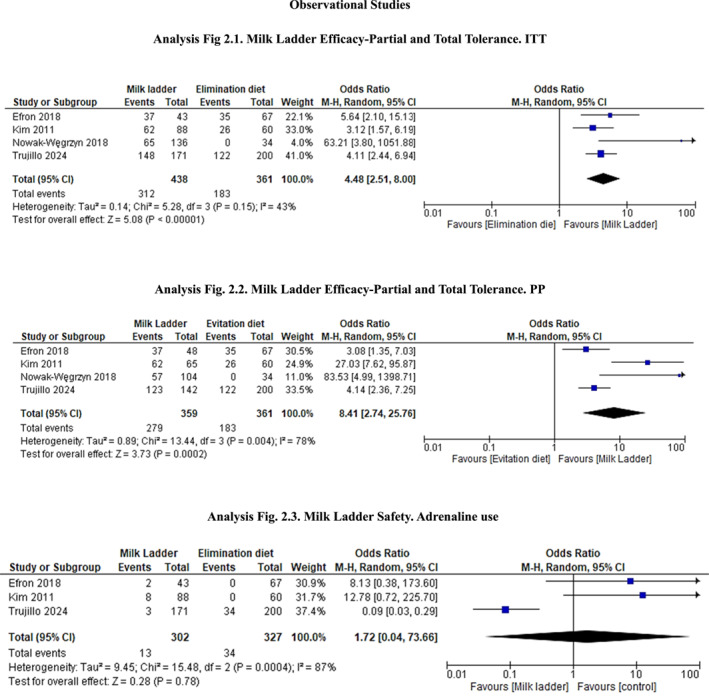
Meta‐analysis.

### 4.4 Methodological Quality

Two studies were conducted in the United States, one in Iran, one in France and one in Israel (Table 2). The follow‐up period for all studies was ≥ 12 months, and the sample ages ranged from 4 months to 17.3 years at baseline. Sample sizes varied from 41 to 371 participants. One randomized controlled study evaluated with the Cochrane tool for assessing risk of bias was of good quality [19]. The quality scores using NOS scales for the selected studies ranged from 5 to 8 points. The studies were of low to medium quality. The primary detection bias was the selection of allergic children based on clinical history and positive skin prick test (SPT) responses or detectable serum milk‐specific IgE without a diagnostic reference test [20, 22]. Only one study used DBPCMC in all patients at the time of enrollment to establish the diagnosis [19]. For the patients to be included in the study, they needed to have a positive history of IgE‐mediated milk allergy that would present with gastrointestinal, cutaneous, respiratory, or systemic manifestations following milk exposure. The allergy of those who met this criterion had then to be confirmed by one of the diagnostic tests. These included milk extract skin prick test (SPT) more than 8 mm or milk sIgE levels (ImmunoCAP, Fisher‐Phadia, Uppsala, Sweden) more than 5 kUA/L in patients with ages < 2 years and more than 15 kUA/L in ones with ages more than 2 years old. Baked milk OFC was performed for all the patients. Those who passed the test were then randomly distributed to the case and control groups; in another case [23], children affected by severe allergic forms were excluded, as inappropriate inclusions could result in overly optimistic estimates of effectiveness and of safety.

Trujillo et al. [24] enrolled patients from Ireland into the active group and patients from Spain into the control group, potentially introducing a geographical bias. The enrollment of two patient cohorts from different countries may introduce a bias due to potential differences in healthcare systems, population demographics, disease management, and data collection methods, thus affecting the internal validity of the study.

The systematic review by Anagnostou et al. presents several flaws and was rated as “critically low” according to AMSTAR 2, due to inadequate response in three critical domains (4, 7, and 11) and one non‐critical domain (10).

Overall, the certainty of the evidence (GRADE) is moderate for all the considered outcomes.

## 5 Discussion

Treatment of CMPA consists of avoidance unless their individual circumstances and risks allow for some consumption. [34] Most children outgrow cow's milk allergy, but the achievement of spontaneous tolerance appears to be slower to develop. The majority (approximately 75%) of children with CMPA tolerate baked milk products [35]. More recently, a randomized clinical study by Esmaeilzadeh et al. [20] showed that ingestion of heated milk in CMPA children who tolerate baked milk accelerates unheated cow's milk tolerance. Therefore, the continuous intake of baked milk can be assimilated to a form of immunotherapy and, according to WAO Guidelines, both can be used as a treatment option for those who have not yet outgrown CMA, but the benefits must be balanced with the adverse effects [35].

ML approach is a third different approach for improving the management of allergic patients. Tolerance can be induced also using the ML method, which involves the step‐by‐step reintroduction of MP, similar to conventional OIT with fresh food, but starting with a food containing cow's milk in a less allergenic form, for example “baked,” then moving through other steps until reaching the gradual introduction of raw cow's milk. For this reason, similarly to OIT, it has been hypothesized that ML may favor tolerance achievement.

The development of tolerance through ladder protocols offers a potential advantage. Venter et al. [7] suggest that the ML method could accelerate the resolution of CM allergy, a finding supported by some controlled studies [20, 22] investigating the impact of repeated baked milk intake on tolerance acquisition. Interest in this method has rapidly grown, since it represents an alternative approach to OIT, being perceived as an easier and more cost‐effective method, reducing healthcare expenses and proving particularly beneficial in settings with limited pediatric allergy resources. One of the main advantages of ML therapy of hospital admission for allergic children and their families, as it can be implemented at home, enhancing children's quality of life, by reducing dietary restrictions. Recently, ML protocols have been published in different countries and adapted to local practices. Various national documents have investigated the ML approach, including BSACI [36], MAP [3], iMAP [7], Mediterranean [37], Indian [38], Canadian [39], German [40], Spanish [25] protocols. Given the increasing interest in this approach, evaluating its advantages and potential drawbacks is crucial, particularly concerning safety aspects requiring careful monitoring by healthcare providers [8].

Regrettably, most of the literature on ML in IgE‐mediated allergic patients comes from observational studies, mostly uncontrolled studies [26] (Table 3). Many children were considered allergic based on medical history and positive allergy tests, while only a few had an OFC‐confirmed diagnosis.

The selection criteria of our systematic review led to the identification of six controlled studies investigating the effectiveness of ML in IgE‐mediated allergic subjects. All studies have shown that the ladder is effective in inducing tolerance earlier than avoidance diet. The meta‐analysis of four studies showed that ML was 4.5 times more effective than the elimination diet in inducing partial or total tolerance in ITT analysis and 8.4 times in PP analysis (Figure 2.1, 2.2).

ML and OIT seems to have a similar effectiveness in tolerance achievement. In the study by Amat et al. [19] tolerance to raw milk was achieved in 36.6% of patients, and partial tolerance was achieved in 26.8%. There was no difference in the gain of tolerance threshold (*p* = 0.24).

The presence of asthma/viral‐induced wheeze had a negative association with the success of the treatment; on the other hand, the lack of recurrent wheezing episodes was associated with successful milk ladder use.

Besides comorbidity, a lower value of milk‐specific IgE was also associated with successful treatments [24].

Regarding ML safety, all the studies included in our systematic review have shown that ML is burdened with some side effects, showing a likelihood of mild adverse reactions in up to 80% of cases in non‐controlled studies, while data from controlled studies are more reassuring (up to 37%). Out of the six studies included in the metanalysis, five studies evaluated ML versus strict elimination diet safety, while only one study, by Amat et al., compared ML to OIT safety [19]. In this study, the step‐up doses were carried out at home for the group treated with the ladder approach, and the incidence of adverse events appeared similar to that observed with the OIT group. The percentage of patients who experienced severe reactions in the two groups was 31.8% and 22.2%, respectively (*p* = 0.72), while the rates for adrenaline use were 14.3% and 11.8%, respectively (*p* = 1). However, this study included all patients referred by their primary allergologist for persistent IgE‐CMA. They were prospectively recruited in the study, regardless of the severity of the allergic reaction (AR), and all patients were characterized by a relatively severe phenotype of CMA. Hence, these results should be cautiously interpreted and cannot be extended to all children with persistent CMA, as the same authors state. In regard to ML safety compared to a strict elimination diet, out of 5 studies, one study did not report data about safety; the results of the study by Kim et al. described five events of mild to moderate anaphylaxis during challenges, but no compared data between the active group (e.g.., the ladder group) versus the control group (strict elimination diet) are reported, while the study by Nowak et al. did not report data about adverse events in the control group (strict elimination diet), stating that 35 percent of AE occurred in the active group, during the baseline‐OFC or escalation‐OFCs in the clinical research center (Table 2).

Hence, the metanalysis of studies reporting adverse events could not be performed due to the small number of studies reporting data on this outcome (*n* = 2).

According to the metanalysis results ML seems to be no different from the avoidance diet for adrenaline use. However, it should be noticed that these results were based only on three studies. Furthermore, the study by Trujillo et al. [24], reporting data on adverse events in the ladder group versus control group as well as adrenaline use, showed that diet seems to be even more burdened by severe allergic reactions than ML. Though this study passed the criteria for being included in our meta‐analysis, it should be pointed out that it is a retrospective study. It included as controls children enrolled in a center of a different nationality (Spanish) than the one (Irish) that used the ladder. The different characteristics of the participants, as correctly recognized by the same authors may have introduced potential confounding variables and unmeasured differences that could have influenced the outcomes.

In summary, the present metanalysis, including, separately, RCT and observational controlled studies, concludes that ML is effective in inducing tolerance earlier than the avoidance diet, with a low risk of bias, in contrast to the results by the metanalysis by Anagnostou et al. [10] This discrepancy may be due to the fact the Anagnostou et al. included both controlled and uncontrolled studies.

In the present systematic review we include randomized controlled trials (RCTs) and observational controlled studies. Though the level of the evidence is different, the inclusion of observational studies is justified since the few existing randomized trials (only two RCTs, involving a total of 125 patients) answer the review question incompletely. However, the inclusion of only controlled observational studies, minimizes the risk of bias, allowing better integration of randomized and non randomized evidence.

The effectiveness of the milk ladder in favoring achievement of tolerance implies avoiding the unnecessary prolongation of a milk‐derivative‐free diet, which in turn can reduce the potential nutritional risk of a prolonged cow's milk avoidance diet, in terms of protein intake and micronutrients deficiencies, common in patients with CM allergy [41, 42]. Furthermore, the psychological impact on patients and families should not be overlooked [43, 44].

Regarding safety, the limited number of studies reporting data on adverse events in the ladder group versus the strict avoidance diet hindered the ability to perform a metanalysis of the adverse events.

Furthermore, heterogeneity exists between different protocols, regarding the setting in which the first dose was administered as well as the numbers of the ladder steps. This implies that the results should be interpreted with caution, especially for which regards the epinephrine use. It should be highlighted that two studies reported the need of adrenaline use at home in the milk ladder group [19, 23]. Considering that severe adverse events have been described, including ones at home, we suggest carrying out the ML with the same precautions that are usually taken when initiating OIT; thus, it is mandatory to provide patients with epinephrine at home.

Where to initiate, when and how to implement the ladder approach still remains debated. In most of the studies considered in the present metanalysis (4 out of 6 studies) the first OFC was conducted in hospital. The incidence of adverse events occurred during the first baseline‐OFC, in children older than 6 years [21, 22]and preschool children [23], though most reactions were mild to moderate, and occurred during the escalation doses, both in the office OFCs [21]and at home [19, 21, 23, 24]^,^.

To use ladders safely in clinical practice, it is important to consider data derived from OIT. A recent phase II randomized, double‐blind, placebo‐controlled trial of BM OIT in persistent CMA phenotype reported that 20% of patients treated with BM required epinephrine during the build‐up phase, confirming the above‐mentioned findings on the ladder approach [45].

We believe that the available data on the safety of administering the first step of the ladder at home in IgE‐mediated cow’s milk allergy are insufficient. In IgE‐mediated CMA, we suggest performing the first baked milk OFC in a hospital setting, under medical supervision, in order to mitigate the risk of severe adverse events. However, depending on local office resources, the choice about where to initiate the milk ladder approach should be left to the physician’s judgment.

Regarding age, the ladder approach is likely to be more suitable for younger children, though the optimal age of ladder implementation has yet to be determined.

The study by Trujllo et al. [24], which considered children with a mean age of 12 months, including those with severe reactions, observed a significantly lower number of severe reactions in children in the ladder group (MAP milk ladder) than the control group (strict elimination diet).

In the study by D'Art et al. [30], which was not included in our systematic review as it did not meet the eligibility criteria, the mean age was lower than all the other studies (< 12 months), authors found that adrenaline was not necessary at home. This would be worthy of note, as lower age was also associated with success of ML in controlled [24] and non‐controlled studies [32], while symptom severity seems to increase with age.

Besides age, the CMA phenotype should also be considered, before starting the milk ladder approach.

Patients at higher risk may be more sensitive to minor changes in protein dose, which will be revealed even among similar types of foods. For this reason, testing for allergenic protein levels may be a useful approach to standardize doses and implementing the ladder safety [46].

As a patient’s sensitization profile may contribute to a different response to the Milk Ladder success, higher IgE levels to milk components may define a high‐risk profile patient who requires caution when implementing the milk ladder approach, since patients with higher IgE levels were demonstrated to be more prone to experience severe reactions during OFC with extensively heated milk [47, 48, 49].

Thus, to implement the ladder approach safely in clinical practice, milk components testing should be done prior to carrying out a ladder approach, in order to allow the physician to be able to make the decision about where and how to implement the ladder approach.

Another open question regards how quickly the patients should move from one step to another.

Based on data of OIT for FA, in high‐risk IgE‐mediated CMA baked milk products ingestion should be considered more cautiously and the time spent on each step should be kept longer; otherwise, the short step intervals may be used in patients with low‐risk IgE‐mediated CMPA. Since adverse reactions are more likely to occur during the starting OFC and the escalation dose, patients should be carefully supervised at each step, mostly during early steps. Thus, we feel that high risk children with IgE mediated CMPA get their up‐dose in hospital under medical supervision, followed by continued ingestion of food products containing equivalent levels of milk proteins at home. If the patient tolerates a sufficient amount of milk product, the gradual increase of the amount at the same step can be continued at home, before moving‐up to the next step.

We believe that this approach may be safer for this CMA phenotype and balances the cost/benefits ratio.

Another potential strategy to minimize patient risks may consist of using baked milk OFC in smaller doses. In the study by Trujllo et al. [24] BM challenge in the active group was performed with doses of MP varied from 0.095 (BM first step) to 0.82 gr MP (BM third step), lower than in other studies, which usually contain ∼0.5–1.3 g of milk protein [19, 20, 22].

Notably, in Trujillo's cohort, 3 out of 32 children (9.3%) in the ladder group experienced anaphylaxis, whereas accidental exposures in the avoidance group led to a much higher rate of anaphylaxis (34/106; 32%; *p* = 1).

Performing BM challenges with low‐doses may play a role in reducing the risks and may be suitable for at high‐risk patients selected for ladder, to reduce the adverse reactions. However, further studies are needed to support these preliminary findings.

Overall, we consider it appropriate to include in ML therapy preschool‐aged children (the younger the better), without asthma, who can tolerate an initial dose of baked milk in a hospital setting. Otherwise, school aged children, with persistent and severe CMA are candidates for OIT.

Since predictive markers for patient selection are currently lacking, due to a few unbiased studies, the selection of patients to treat with milk ladder approach should be based on clinical judgment and expertise and should be carefully evaluated in specialized centers. Children who did not tolerate even trace amounts of baked milk, those with a history of severe adverse reactions at onset and/or high levels of sIgE, and children diagnosed with asthma, especially if not controlled, should be excluded from this approach. The findings of the present metanalysis are relevant to all professionals involved in CMA management, including nurses, nurse practitioners and dietitians, who contribute in patient education and dietary supervision.

## 6 Conclusion

In recent years, several studies on ML have been conducted: the availability of evidence now necessitates drawing conclusions and making recommendations based on systematic reviews rather than on expert opinion. Our Systematic Review demonstrates the efficacy of ML in children in favoring tolerance achievement, compared to a strict diet avoidance. As for safety, a meta‐analysis of three studies shows that safety of ML does not seem significantly different from the avoidance diet, in terms of adrenaline use. These conclusions, however, are limited by the small number of studies and by the lack of robust, high‐quality meta‐analyzed data. Further research is needed in order to clarify milk ladder safety, as well as to identify predictive biomarkers for patient selection.

## Author Contributions

**Barbara Cuomo:** conceptualization, methodology, formal analysis, investigation, writing – original draft, writing – review and editing. **Maria Carmen Verga:** conceptualization, methodology, validation, formal analysis, investigation, resources, writing – original draft, writing – review and editing. **Enza D’Auria:** writing – original draft, writing – review and editing, supervision. **Michele Miraglia Del Giudice:** supervision. **Caterina Anania:** writing – original draft. **Fabio Decimo:** writing – original draft. **Giuliana Gianni:** writing – original draft. **Giovanni Cosimo Indirli:** writing – original draft. **Enrica Manca:** writing – original draft. **Filippo Mondi:** investigation, writing – original draft. **Valentina Nosratian:** writing – original draft. **Erica Pendezza:** writing – original draft. **Marco Ugo Andrea Sartorio:** writing – original draft. **Mauro Calvani:** validation, investigation, writing – review and editing.

## Funding

The authors have nothing to report.

## Ethics Statement

Formal ethics approval is unnecessary since no private information about the participants will be involved.

## Conflicts of Interest

The authors declare no conflicts of interest.

## Supporting information


Supporting Information S1

Supporting Information S2

Supporting Information S3

## Data Availability

The data that support the findings of this study are available in the supplementary material of this article Supporting Information [Link], [Link], [Link].
