# Supplementary material for: Milk Ladder Efficacy and Safety in IgE‐Mediated Cow's Milk Allergy: A Systematic Review and Meta‐Analysis of Controlled Studies
Source: Clin Transl Allergy. 2025 Nov 28;15(12):e70122. doi: 10.1002/clt2.70122 (PMC12661123; doi:10.1002/clt2.70122)
Supplement: Supplementary file 1 — Supporting Information S1 [file CLT2-15-e70122-s002.docx]

**Systematic Reviews Search**

Temporal Limitation: from January 1, 2000, through December 15, 2024

**PUBMED**

#1

("milk allergy"[All Fields] OR "food allergy"[All Fields]) AND ("baked milk"[All Fields] OR "processed milk"[All Fields] OR "cooked milk"[All Fields] OR (("hot temperature"[MeSH Terms] OR ("hot"[All Fields] AND "temperature"[All Fields]) OR "hot temperature"[All Fields] OR "heat"[All Fields]) AND ("modifiable"[All Fields] OR "modified"[All Fields] OR "modifier"[All Fields] OR "modifiers"[All Fields] OR "modifies"[All Fields] OR "modify"[All Fields] OR "modifying"[All Fields]) AND ("milk, human"[MeSH Terms] OR ("milk"[All Fields] AND "human"[All Fields]) OR "human milk"[All Fields] OR "milk"[All Fields] OR "milk"[MeSH Terms])) OR "extensively heated milk"[All Fields] OR "heated milk"[All Fields]) AND ("ladder"[All Fields] OR "tolerance"[All Fields] OR "desensitisation"[All Fields] OR "immunotherapy"[All Fields] OR "safety"[All Fields]) AND ( [cochrane AND review] OR [systematic AND review] OR [meta AND analysis] )

**SCOPUS**

#1

TITLE-ABS-KEY ( ( "milk allergy" OR "food allergy") AND ( "baked milk" OR "processed milk" OR "cooked milk" OR ( ( "hot temperature" OR ( "hot" AND "temperature") OR "hot temperature" OR "heat" ) AND ( "modifiable" OR "modified" OR "modifier" OR "modifiers" OR "modifies" OR "modify" OR "modifying") AND ( "milk, human" OR ( "milk" AND "human") OR "human milk" OR "milk" OR "milk" ) OR "extensively heated milk" OR "heated milk" ) AND ( "ladder" OR "tolerance" OR "desensitisation" OR "immunotherapy" OR "safety") ) AND ( [cochrane AND review] OR [systematic AND review] OR [meta AND analysis] )

**EMBASE:**

( 'milk allergy' OR 'food allergy' ) AND ( 'baked milk' OR 'processed milk' OR 'cooked milk' OR ( ( 'hot temperature' OR ( 'hot' AND 'temperature' ) OR 'heat' ) AND ( 'modifiable' OR 'modified' OR 'modifier' OR 'modifiers' OR 'modifies' OR 'modify' OR 'modifying' ) AND ( 'milk, human' OR ( 'milk' AND 'human' ) OR 'human milk' OR 'milk' ) OR 'extensively heated milk' OR 'heated milk' ) ) AND ( 'ladder' OR 'tolerance' OR 'desensitisation' OR 'immunotherapy' OR 'safety' ) AND ( 'cochrane review' OR 'systematic review' OR 'meta analysis' )

**COCHRANE**

("milk allergy" OR "food allergy") AND ("baked milk" OR "processed milk" OR "cooked milk" OR "heated milk" OR "extensively heated milk" OR (("hot temperature" OR heat) AND (modifiable OR modified OR modifier OR modifiers OR modifies OR modify OR modifying) AND ("human milk" OR milk))) AND (ladder OR tolerance OR desensitisation OR immunotherapy OR safety) AND ("systematic review" OR "Cochrane review" OR "meta-analysis")
