# Supplementary material for: Milk Ladder Efficacy and Safety in IgE‐Mediated Cow's Milk Allergy: A Systematic Review and Meta‐Analysis of Controlled Studies
Source: Clin Transl Allergy. 2025 Nov 28;15(12):e70122. doi: 10.1002/clt2.70122 (PMC12661123; doi:10.1002/clt2.70122)
Supplement: Supplementary file 2 — Supporting Information S2 [file CLT2-15-e70122-s003.docx]

**S2. MILK LADDER**

**METHODOLOGICAL ASSESSMENT OF THE SRs AND STUDIES**

| **Table S2.1. SR Appraisal** | |
| --- | --- |
| **AMSTAR 2** | Anagnostou et al. 2024 |
| **1.**  **Did the research questions and inclusion criteria for the review include the components of PICO?**  **(Yes/No)** | Yes |
| **2.**  **Did the review report explicitly state that the review methods were established before the review, and did the report justify any significant deviations from the protocol?**  **(Yes/Partial Yes/No)** | Yes |
| **3. Did the review authors explain their selection of the study designs for inclusion in the review?**  **(Yes/No)** | Yes |
| **4. Did the review authors use a comprehensive literature search strategy?**  **(Yes/Partial Yes/No)** | Partial Yes |
| **5.**  **Did the review authors perform study selection in duplicate?**  **(Yes/No)** | Yes |
| **6. Did the review authors perform data extraction in duplicate?**  **(Yes/No)** | Yes |
| **7. Did the review authors provide a list of excluded studies and justify the exclusions?**  **(Yes/Partial Yes/No)** | No |
| **8. Did the review authors describe the included studies in adequate detail?**  **(Yes/Partial Yes/No)** | Partial Yes |
| **9. Did the review authors use a satisfactory technique for assessing the risk of bias (RoB) in individual studies included in the review?**  **(Yes/Partial Yes/No/Includes only NRSI-RCT)** | Yes |
| **10. Did the review authors report on the funding sources for the studies included in the review?**  **(Yes/No)** | No |
| **11. If meta-analysis was performed, did the review authors use appropriate methods for combining statistical results?**  **(Yes / No / No meta-analysis conducted)** | No |
| **12. If meta-analysis was performed, did the review authors assess the potential impact of RoB in individual studies on the results of the meta-analysis or other evidence synthesis?**  **(Yes / No / No meta-analysis conducted)** | Yes |
| **13. Did the review authors account for RoB in individual studies when interpreting/ discussing the results of the review?**  **(Yes/No)** | Yes |
| **14. Did the review authors provide a satisfactory explanation for and discussion of any heterogeneity observed in the results of the review?**  **(Yes/No)** | Yes |
| **15. If they performed quantitative synthesis, did the review authors carry out an adequate investigation of publication bias (small study bias) and discuss its likely impact on the results of the review?**  **(Yes / No / No meta-analysis conducted)** | Not Applicable |
| **16. Did the review authors report any potential sources of conflict of interest, including any funding they received for conducting the review?**  **(Yes/No)** | Yes |
| **OVERALL EVALUATION**  **CRITICAL DOMAINS #2, 4, 7, 9, 11, 13, 15**  *** Presence of 2 critical items (n. 7, 11) and 1 non-critical item failed (n. 10)** | **Critically Low*** |

**Table S2.2. Studies Appraisal - Cohort Studies.**

|  | **Newcastle Quality Assessment Scale**  **COHORT STUDIES** | | |  |  |  |  |  | |  |  |
| --- | --- | --- | --- | --- | --- | --- | --- | --- | --- | --- | --- |
|  | **Selection** |  |  |  | **Comparability** | **Outcome** |  | |  | |  |
| **Study** | **Representativeness of the exposed cohort** | **Selection of the non-exposed cohort** | **Ascertainment of exposure** | **Demonstration that outcome of interest was not present at the start of the study** | **Comparability of cohorts based on the design or analysis** | **Assessment of outcome** | **Was follow-up long enough for outcomes to occur?** | | **Adequacy of follow-up of cohorts** | | **Total** |
| Kim et al. 2011 | 0c | 0c | 1a | 1a | 0c | 0c | 1a | | 1a | | 5/9 |
| Nowak-Węgrzyn et al. 2018 | 0c | 0c | 1a | 1a | 2 a,b | 0c | 1a | | 1a | | 8/9 |
| Trujillo et al. 2024 | 0c | 0c | 1a | 1a | 1a | 1b | 1a | | 1a | | 6/9 |

**Table S2.3. Studies Appraisal - Case-Control Studies.**

|  | **Newcastle Quality Assessment Scale** | | |  |  |  |  | | | |
| --- | --- | --- | --- | --- | --- | --- | --- | --- | --- | --- |
|  | **CASE-CONTROL STUDIES** | | |  |  |  |  | | | |
|  | **Selection** |  |  |  | **Comparability** | **Exposure** |  |  |  |  |
| **Study** | **Adequate case definition** | **Case Representativeness** | **Selection of Controls (community)** | **Definition of Controls (no outcome)** | **Comparability of cases and controls based on the design or analysis.** | **Ascertainment of exposure** | **The same method of ascertainment for cases and controls** | **Non-Response rate** | **Total** |  |
| Efron et al. 2018 | 0b | 0b | 1a | 1a | 2ab | 1a | 1a | 1a | 6/8 |  |

**RANDOMISED CLINICAL TRIALS (RCTs)**

**Figure S2.1. Risk of bias summary: review authors' judgments about each risk of bias item for each included study.**


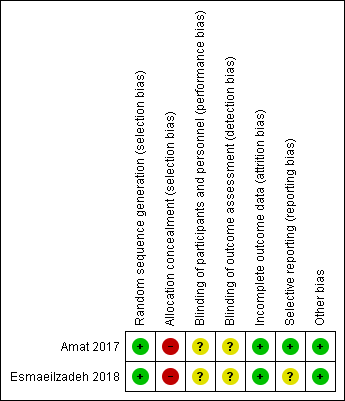


**Figure S2.2. Risk of bias graph: review authors' judgments about each risk of bias item presented as percentages across all included studies.**


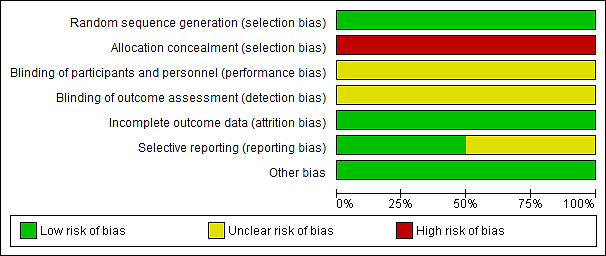


| **Table S2.4. Excluded studies with reasons for exclusion.** | |
| --- | --- |
| **Excluded studies** | **Reason for exclusion** |
| Cerecedo 2024 | Not controlled study |
| Gallagher 2024 | Data extracted from Heng’s study 2023 [ ] |
| Chomyn 2024 | Non-controlled study |
| Cronin et al. 2023 | Non-controlled study |
| Heng et al. 2023 | Non-controlled study |
| D’Art et al. 2022 | All children undergo ladder therapy. Data were reported from a group of children who tolerated a supervised low-dose of 0.5 mg of fresh milk, and from a second group starting with a ladder scale at home without a tolerated starting dose under medical supervision |
| Ball et al. 2019 | Non-controlled study |
| Dunlop et al. 2018 | Non-controlled study.  All children undergo ladder therapy. Data were reported from a group of children who tolerated a supervised starting dose of 2 g of baked milk and started the well-defined ladder therapy. They were also from a second group that did not pass the supervised tolerance test and sent home with instructions to include some amount of BM in  their diet. |
| Weinbrand-Goichberg et al. 2017 | Non-controlled study |
