# Supplementary material for: Milk Ladder Efficacy and Safety in IgE‐Mediated Cow's Milk Allergy: A Systematic Review and Meta‐Analysis of Controlled Studies
Source: Clin Transl Allergy. 2025 Nov 28;15(12):e70122. doi: 10.1002/clt2.70122 (PMC12661123; doi:10.1002/clt2.70122)
Supplement: Supplementary file 3 — Supporting Information S3 [file CLT2-15-e70122-s001.docx]

**S3. SUMMARY OF FINDINGS FOR THE MAIN COMPARISON**

**Table S3.1. Milk Ladder Efficacy-Partial and Total Tolerance**

| **[Milk Ladder] compared to [Elimination diet] for [Cow Milk Allergy. Partial and Total Tolerance]. ITT Analysis** | | | | | |
| --- | --- | --- | --- | --- | --- |
| **Patient or population:** [Cow Milk Allergy. Partial and Total Tolerance]  **Setting:** Outpatient  **Intervention:** [Milk Ladder]  **Comparison:** [Elimination diet] | | | | | |
| **Outcomes** | **№ of participants (studies) Follow-up** | **Certainty of the evidence (GRADE)** | **Relative effect (95% CI)** | **Anticipated absolute effects** | |
|  |  |  |  | **Risk with [Elimination diet]** | **Risk difference with [Milk Ladder]** |
| Milk Ladder Efficacy-Partial and Total Tolerance_Observational Studies (ML Efficacy_OSs) assessed with: % of patients achieving total or partial tolerance to CMP follow-up: range 6 months to 12 months^1,2,3,4^ | 799 (4 non-randomised studies)^1,2,3,4^ | ⨁⨁⨁◯ Moderate^1,2,3,4, a,b^ | **OR 4.48** (1.51 to 8.00) | 507 per 1.000 | **315 more per 1.000** (214 more to 385 more) |
| Milk Ladder Efficacy-Partial and Total Tolerance_RCT (ML Efficacy_RCT) assessed with: % of patients achieving total or partial tolerance to CMP follow-up: mean 12 months | 84 (1 RCT)^5^ | ⨁⨁⨁◯ Moderate^c^ | **RR 1.32** (1.04 to 1.68) | 667 per 1.000 | **213 more per 1.000** (27 more to 453 more) |
| ***The risk in the intervention group** (and its 95% confidence interval) is based on the assumed risk in the comparison group and the **relative effect** of the intervention (and its 95% CI).  **CI:** confidence interval; **RR:** risk ratio | | | | | |
| **GRADE Working Group grades of evidence** **High certainty:** we are very confident that the true effect is close to the effect estimate's. **Moderate certainty:** we are moderately confident in the effect estimate: the true effect is likely to be close to the estimate of the effect, but there is a possibility that it is substantially different. **Low certainty:** our confidence in the effect estimate is limited: the true effect may be substantially different from the estimate of the effect. **Very low certainty:** we have very little confidence in the effect estimate: the true effect is likely to be substantially different from the estimate of effect. | | | | | |

#### Explanations

a. The control group didn't derive from the same population

b. Diagnosis made without OFC

c. Selection bias

#### References

1. Efron, et al. .2018.

2. Kim, et al..2011.

3.Nowak-Węgrzyn,et al. .2018 .

4. Trujlo et al.

5. Esmaeilzadeh,et al. .2018.

**Table S3.2. Milk Ladder vs. OIT Efficacy-Partial and Total Tolerance**

| **[Milk Ladder] compared to [Elimination diet] for [Cow Milk Allergy. Partial and Total Tolerance]** | | | | | |
| --- | --- | --- | --- | --- | --- |
| **Patient or population:** [Cow Milk Allergy. Partial and Total Tolerance]  **Setting:** Outpatient  **Intervention:** [Milk Ladder]  **Comparison:** [Elimination diet] | | | | | |
| **Outcomes** | **№ of participants (studies) Follow-up** | **Certainty of the evidence (GRADE)** | **Relative effect (95% CI)** | **Anticipated absolute effects** | |
|  |  |  |  | **Risk with [Elimination diet]** | **Risk difference with [Milk Ladder]** |
| Milk Ladder vs. OIT Efficacy-Partial and Total Tolerance_RCT (Milk Ladder vs. OIT Efficacy) (Milk Ladder vs. OIT Efficacy) Assessed with: Mean Difference in ml of CMP tolerated follow-up: mean 18 months (ML vs.OIT) assessed with: Mean Difference in ml of CMP tolerated MD (95% IC) follow-up: mean 18 months^1^ | 41 (1 RCT)^6^ | ⨁⨁⨁◯ Moderate^a^ | The average daily ingested dose of MP was 1258 mg [476-2720] in the high-risk arm vs 714 mg [106-2720] in the low-risk arm (P= 0.24). | | |
| ***The risk in the intervention group** (and its 95% confidence interval) is based on the assumed risk in the comparison group and the **relative effect** of the intervention (and its 95% CI).  **CI:** confidence interval; **OR:** odds ratio; **RR:** risk ratio | | | | | |
| **GRADE Working Group grades of evidence** **High certainty:** we are very confident that the true effect lies close to that of the estimate of the effect. **Moderate certainty:** we are moderately confident in the effect estimate: the true effect is likely to be close to the estimate of the effect, but there is a possibility that it is substantially different. **Low certainty:** our confidence in the effect estimate is limited: the true effect may be substantially different from the estimate of the effect. **Very low certainty:** we have very little confidence in the effect estimate: the true effect is likely to be substantially different from the estimate of effect. | | | | | |

#### Explanations

c. d. Selection bias

#### References

6. Amat, et al..2017.

**Table S3.3. Milk Ladder Safety**

| **[Milk Ladder] compared to [Elimination diet] for [Cow Milk Allergy. Safety]** | | | | | |
| --- | --- | --- | --- | --- | --- |
| **Patient or population:** [Cow Milk Allergy. Safety]  **Setting:** Outpatient  **Intervention:** [Milk Ladder]  **Comparison:** [Elimination diet] | | | | | |
| **Outcomes** | **№ of participants (studies) Follow-up** | **Certainty of the evidence (GRADE)** | **Relative effect (95% CI)** | **Anticipated absolute effects** | |
|  |  |  |  | **Risk with [Elimination diet]** | **Risk difference with [Milk Ladder]** |
| Milk Ladder vs. Elimination diet. Safety (ML Safety) assessed with: % Patients requiring epinephrine use follow-up: range 6 months to 12 months | 481 (2 non-randomised studies)^1,2^ | ⨁⨁⨁◯ Moderate^a,b,c^ | **OR 3.61** (0.00 to 3986.62) | 397 per 1.000 | **307 more per 1.000** (397 fewer to 603 more) |
| Milk Ladder Safety. Adrenaline used (ML Safety_Adrenaline) | 629 (3 non-randomised studies)^1,2,3^ | ⨁⨁⨁◯ Moderate^a,b,c^ | **OR 1.72** (0.04 to 73.66) | 104 per 1.000 | **62 more per 1.000** (99 fewer to 791 more) |
| ***The risk in the intervention group** (and its 95% confidence interval) is based on the assumed risk in the comparison group and the **relative effect** of the intervention (and its 95% CI).  **CI:** confidence interval; **RR:** risk ratio | | | | | |
| **GRADE Working Group grades of evidence** **High certainty:** we are very confident that the true effect lies close to that of the effect estimate. **Moderate certainty:** we are moderately confident in the effect estimate: the true effect is likely to be close to the estimate of the effect, but there is a possibility that it is substantially different. **Low certainty:** our confidence in the effect estimate is limited: the true effect may be substantially different from the estimate of the effect. **Very low certainty:** we have very little confidence in the effect estimate: the true effect is likely to be substantially different from the estimate of effect. | | | | | |

#### Explanations

a. The control group didn't derive from the same population

b. Diagnosis made without OFC

c. High 95% IC

#### References

1. Kim, et al. .2011.

2. Efron, et al. .2018.

3. Trujllo et al. 2024

**Table S3.4. Milk Ladder vs. OIT. Safety**

| **[Milk Ladder] compared to [OIT] for [Cow Milk Allergy. Safety]** | | | | | |
| --- | --- | --- | --- | --- | --- |
| **Patient or population:** [Cow Milk Allergy. Safety]  **Setting:** Outpatient  **Intervention:** [Milk Ladder]  **Comparison:** [OIT] | | | | | |
| **Outcomes** | **№ of participants (studies) Follow-up** | **Certainty of the evidence (GRADE)** | **Relative effect (95% CI)** | **Anticipated absolute effects** | |
|  |  |  |  | **Risk with [OIT]** | **Risk difference with [Milk Ladder]** |
| Milk Ladder vs. OIT Safety (Milk Ladder vs. OIT Safety) assessed with: % Patient experiencing at least one AR follow-up: mean 18 months | 41 (1 RCT)^1^ | ⨁⨁⨁◯ Moderate^a^ | **RR 0.85** (0.50 to 1.46) | 667 per 1.000 | **100 fewer per 1.000** (333 fewer to 307 more) |
| Milk Ladder vs. OIT Safety_Adrenaline use (ML vs. OIT Safety_Adrenaline use) assessed with: % Patients requiring epinephrine use follow-up: mean 18 months | 41 (1 RCT)^1^ | ⨁⨁⨁◯ Moderate^a^ | **RR 1.17** (0.22 to 6.30) | 167 per 1.000 | **28 more per 1.000** (130 fewer to 883 more) |
| ***The risk in the intervention group** (and its 95% confidence interval) is based on the assumed risk in the comparison group and the **relative effect** of the intervention (and its 95% CI).  **CI:** confidence interval; **RR:** risk ratio | | | | | |
| **GRADE Working Group grades of evidence** **High certainty:** we are very confident that the true effect lies close to that of the effect estimate. **Moderate certainty:** we are moderately confident in the effect estimate: the true effect is likely to be close to the estimate of the effect, but there is a possibility that it is substantially different. **Low certainty:** our confidence in the effect estimate is limited: the true effect may be substantially different from the estimate of the effect. **Very low certainty:** we have very little confidence in the effect estimate: the true effect is likely to be substantially different from the estimate of effect. | | | | | |

#### Explanations

a. Selection bias

#### References

1. Amat, et al .2017.
